# Responsible governance of human germline genome editing in China^†^

**DOI:** 10.1093/biolre/ioac114

**Published:** 2022-05-27

**Authors:** Yaojin Peng, Jianwei Lv, Lulu Ding, Xia Gong, Qi Zhou

**Affiliations:** State Key Laboratory of Stem Cell and Reproductive Biology, Institute of Zoology, Chinese Academy of Sciences, Beijing, China; Institute for Stem Cell and Regeneration, Chinese Academy of Sciences, Beijing, China; University of the Chinese Academy of Sciences, Beijing, China; Beijing Institute for Stem Cell and Regenerative Medicine, Beijing, China; State Key Laboratory of Stem Cell and Reproductive Biology, Institute of Zoology, Chinese Academy of Sciences, Beijing, China; Institute for Stem Cell and Regeneration, Chinese Academy of Sciences, Beijing, China; State Key Laboratory of Stem Cell and Reproductive Biology, Institute of Zoology, Chinese Academy of Sciences, Beijing, China; Institute for Stem Cell and Regeneration, Chinese Academy of Sciences, Beijing, China; State Key Laboratory of Stem Cell and Reproductive Biology, Institute of Zoology, Chinese Academy of Sciences, Beijing, China; Institute for Stem Cell and Regeneration, Chinese Academy of Sciences, Beijing, China; State Key Laboratory of Stem Cell and Reproductive Biology, Institute of Zoology, Chinese Academy of Sciences, Beijing, China; Institute for Stem Cell and Regeneration, Chinese Academy of Sciences, Beijing, China; University of the Chinese Academy of Sciences, Beijing, China

**Keywords:** human germline genome editing, ethics, public debates, governance, China

## Abstract

Considerable improvements have been made to gene editing technology, which has been increasingly applied to research involving humans. Nevertheless, human heritable germline genome editing is associated with a series of potential ethical, legal, and social risks, which have generated major controversies and discussions worldwide, especially after the “gene-edited babies” incident. Influenced by this incident, China has realized the importance of ethical governance in the field of life science and technology, has accelerated legislative and policy efforts in this field, and has gradually moved toward the direction of “precautionary” ethical governance. Black letter analysis, big data public opinion analysis, and other research methods are used in this paper. This paper explores the scientific background, ethical debates, and latest developments regarding China’s regulatory framework for human germline gene editing after the “gene-edited babies” controversy and provides several recommendations on the future governance system of human germline gene editing in China. This paper argues that in recent years, the ethics governance of germline genome editing in China has been accelerated and great changes have been made. However, the regulatory system for germline genome editing requires further improvement in three aspects: coordination of legislation and agencies, establishment of an ethics review system at high levels, and public participation and education.

## Teaser

Continual progress has been made in establishing a governance system of ethical issues regarding human genome editing, particularly human germline genome editing; however, further improvements are required.

## Graphical Abstract

**Graphical Abstract:**
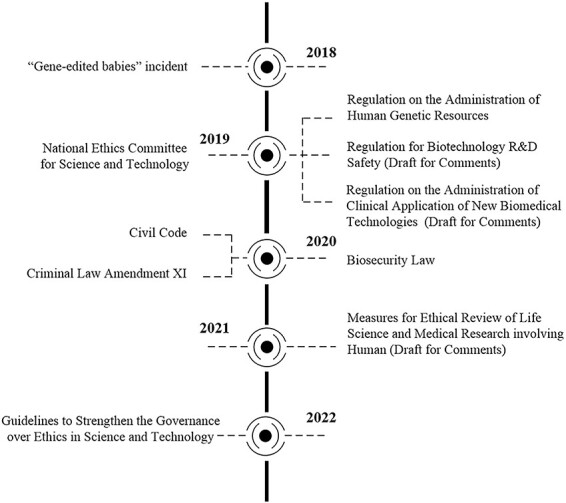


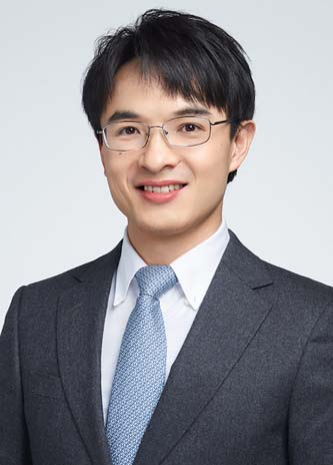


Dr. Yaojin Peng is Zhiyi Professor (co-appointed) at the Beijing Institute of Stem Cell and Regenerative Medicine (BISCRM) and theInstitute of Zoology (Chinese Academy of Sciences); and Director of the Centre for Ethics of Science and Technology (CEST) of the BISCRM. Dr. Peng holds a Bachelor’s degree in life science and a Master’s degree in Law. He finished his PhD at the Faculty of Law, Maastricht University, the Netherlands. Dr. Peng’s research focuses on biotechnology law and bioethics, intellectual property rights (patents) and standards, science and technology policy and management. Dr. Peng has published more than 20 articles in international top academic journals, such as Nature Biotechnology (2x), Cell Stem Cell, Protein & Cell, etc. He currently is a member of the expert committee of China Stem Cell and Regenerative Medicine Collaborative Innovation Platform; Deputy Secretary General of the Academic Committee of the Center for Science & Technology Development and Governance, Tsinghua University; a member of the Standard Working Group of Chinese Society for Stem Cell Research (CSSCR). Dr. Peng has received numerous academic awards, such as the 2021 Youth Innovation Promotion Association of the Chinese Academy of Sciences, and 2021 Advanced Individual for Stem Cell Social Welfare of the CSSCR.

## Introduction

In recent years, gene editing technology, in particular the new genetic engineering tool known as clustered regularly interspaced short palindromic repeats (CRISPR) [1–3], has enabled human genomic research worldwide; this new tool is much cheaper, more efficient, and more accurate than traditional gene editing techniques [4–6]. Advances in genetics provide hope in terms of the possibility of curing major genetic diseases [7–10]. Gene editing technologies, however, have created considerably controversial ethical, social, and legal issues [11], in particular because these technologies have increasingly targeted human gametes or embryos recently. For instance, in April 2015, a research group revealed that they used CRISPR-Cas9 to genetically modify nonviable, triploid embryos [12, 13], which led to fierce international debates [14–16].

On 26 November 2018, a scientist declared to have generated the world’s first genetically edited babies [17]. This news once again triggered debates on the safety, ethical, and social issues related to gene editing technologies worldwide [18] and reignited discussions on the appropriate regulation of germline genome editing [19]. Human germline genome editing has gained prominence in public discourse, and the “gene-edited babies” incident highlighted the substantial social and ethical hazards associated with human germline gene editing, especially in jurisdictions with a regulatory vacuum.

China has accelerated the drafting and revision of laws or regulations and has introduced several measures to strengthen the ethical governance of science and technology. In the field of life science and technology, especially concerning human germline genome editing, a comprehensive exploration of policy and legislative changes in China as well as pertinent implications is vital. In this regard, the paper examines the scientific background, ethical debates, and China’s regulatory framework regarding human germline genome editing as well as recent developments in the relevant regulations, particularly after the aforementioned incident, and the potential implications of these changes. This paper concludes by proposing several recommendations for China’s future governance system concerning human germline genome editing.

## Rise of scientific and ethical debates

Views differ on whether human germline genome editing should be used. The most frequent justification for the clinical application of human germline genome editing is that the technology can help parents with severe genetic diseases avoid passing these diseases on to their babies [20]. Moreover, it is believed that human germline genome editing can be used to reduce the risk of common diseases, such as cancer, diabetes, heart disease, and multiple sclerosis [21], and may even introduce certain rare genetic features or enhance others to improve human capabilities, such as prolonging life span, improving intelligence quotient, increasing muscle strength, and changing emotional control ability [22]. For these reasons, some individuals argue that germline genome editing is an inevitable part of humanity’s future [23].

## Debates concerning the scientific risks

The aforementioned advantages of human germline genome editing are, however, based on the optimistic notion of smooth science and technology development. The reality is that considerable technological risks exist in germline genome editing. One concern is that the gene editing technologies, including CRISPR, do not meet the extremely high accuracy and precision required for germline editing. Off-target mutagenesis and mosaicism can be substantial drawbacks of gene editing technologies [24], particularly in terms of clinical applications; nevertheless, off-target effects appear to be sufficiently rare to enable most research applications [25]. For instance, a surprising number of “off-target” mutations were found in the first CRISPR-Cas9 experiments on nonviable embryos in China [12]. Moreover, a comprehensive understanding is yet to be gained of the relationship between genes and complex features of the human body [20]; even in the absence of off-target effects, intended gene edits might cause unintended consequences [20, 26]. For example, research has revealed that gene-edited babies may be at higher risk of death [27]. Thus, many scientific challenges regarding human germline genome editing remain to be overcome. These scientific concerns will likely be solved in the future with the development of science and technology [14].

## Debates regarding ethical and social issues

Human germline genome editing can have adverse social consequences, and this technology exhibits considerable ethical complexity. One major social or ethical concern is related to eugenics or neoeugenics. Germline genome editing can be used to introduce or enhance certain rare genetic features of humans; hence, the use of this technique might change social value and culture by eroding the instincts for the unconditional acceptance of differences or imperfections of people, such as disabilities [6]; this is likely to reduce the public’s tolerance for “unfit” traits or conditions of individuals and to reinforce prejudice as well as narrow the definitions of normalcy in society [6]. Another major social or ethical concern is related to social justice and equality. The use of human germline genome editing may perpetuate existing inequities within societies and exacerbate social hierarchy [28]. In particular, in the initial stage of market entry, only those in certain geographic locations with considerable financial resources would be able to use genetic editing technology to “change” their children. In that case, elites would be able to increase their advantages, whereas the civilian class would be unable to change its “deficiencies” [6].

**Figure 1:**
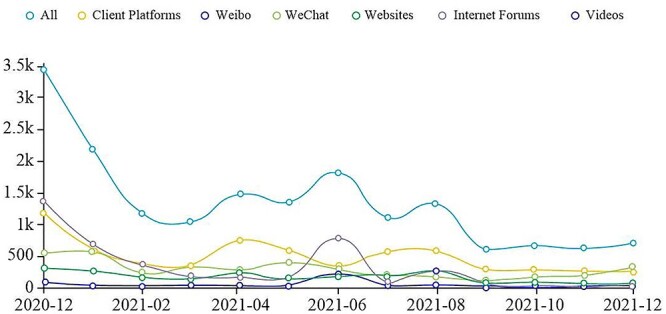
Information transmission trend.

In addition, many people express the concern that the dependence on human germline genome editing may obscure the important topic of social mechanism reform. Such editing may trigger excessive attention toward individuals, because this technology focuses on manipulation at the individual level, rather than at a broader group or social level [29]. This overemphasis on altering individuals’ genome may lead the public to believe that genetic modification is more promising and important in terms of improving human life than are social environmental approaches [6]. Furthermore, other ethical concerns exist, such as those related to intergenerational informed consent, as germline genome edits are heritable [6], and those related to human dignity based on the manipulation of embryos or gametes. These ethical concerns are not limited to human germline genome editing but are more broadly related to gene therapy in general.

## Recent public debates in China

Recently, especially since the “gene-edited babies” incident at the end of 2018, ethical, legal, and social issues regarding human germline gene editing have been widely discussed in China by not only those in academia but also the public. We used “gene-edited babies,” “human embryo gene editing,” and “human reproductive line gene editing” as the key words to conduct a search on the big data public opinion analysis platform of Sina Yuqingtong (https://yqt.mdata.net/) to analyze Chinese public debate in the past year (from 1 December 2020 to 23 December 2021).^1^

According to the results, the total number of messages on this topic is 17 523, with a peak value of 3466 and an average propagation speed of 1460.25 messages/month. In terms of the information transmission trend, the development trend of public opinion is moderate. The peak of the whole event was in December 2020, with considerable attention paid by the media and netizens to this topic. Subsequently, the debate gradually diminished (Figure 1).

On one hand, the frequency of popular words in the media can be seen as follows, “ethics,” “risk,” “gene editing,” “embryo,” and “law” are all in the top 10 (see Table 1). This shows the public’s concern about the ethical risks of human germline gene editing and hopes that laws and regulations will keep pace with the development of technological progress to regulate such disruptive and pioneering technologies.

**Figure 2:**
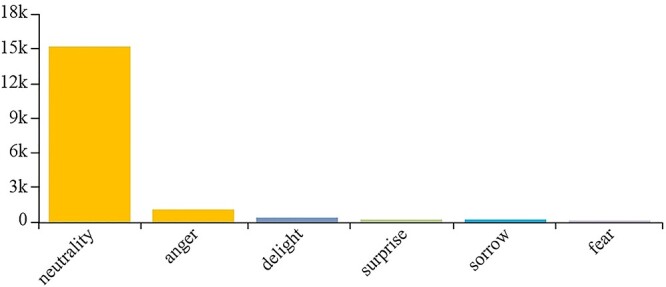
Public’s emotional response to related events over 1 year.

On the other hand，according to our big data analysis, the public’s emotional response to related events over 1 year (Figure 2) was as follows: neutral emotions (no = 15 342; 87.55%), anger (no = 1220; 6.96%), and joy (no = 380; 2.17%); the public has a relatively objective neutral mood overall and still has expectations for scientific and technological progress.

**Table 1:** Frequency of popular words in the media

No.	Hot words	Frequency
1	Mankind	1813
2	Face recognition	1547
3	Ethics	1519
4	Research	1237
5	Technology	1122
6	Risk	987
7	Genome editing	878
8	Gene	807
9	Embryo	736
10	Law	735

In general, the Chinese public and scholars have substantial ethical concerns regarding human germline genome editing. Moreover, they are concerned about the current situation of gene-edited babies and the results of subsequent treatment, and they hope that the government can promptly establish relevant legislation for the life science and technology field.

Considering the potential scientific risks and complex ethical issues, the possibility for abuse of the technology, and the extensive public attention, genome editing of human embryos is premature and should be cautiously undertaken under restrictive regulations [30]; this is also true for the clinical applications of germline genome editing; scientist have repeatedly called for a moratorium on clinical applications [31]. Moreover, a strong and clear regulatory environment is helpful for promoting investment and innovation, as well as the production and sale of high-quality products and technologies in the biotechnology industry [32].

## Recent development of regulation in China

In China, basic research on gene therapy began in the 1980s. Since then, the Chinese government has attached great importance to policies and regulations for embryo research and gene therapy and has also continually improved the relevant regulatory framework. In general, prior to the “gene-edited babies” incident, laws, or regulations that directly address human germline genome editing were absent in China. Provisions related to this technology were distributed across several department rules and regulatory documents promulgated by various ministries and commissions under the State Council, such as the Ministry of Science and Technology (MOST) and National Health Commission (NHC), which directly or indirectly regulate embryo or embryonic stem cell research, research using gene editing, in vitro fertilization therapy, or research and clinical trials on human subjects. However, although most of these provisions could regulate R&D and development in the life science field, these provisions had not kept pace with the development of science and technology, especially considering the rapid development of gene editing technology, synthetic biology technology, and stem cell therapy in the recent decade.

Over recent years, especially since the “gene-edited babies” incident, the Chinese government has attached great importance to the governance of biotechnology and its applications. For instance, in October 2019, China established the National Science and Technology Ethics Committee (NSTEC), with three subcommittees, including the Life Science Ethics Sub-Committee. In March 2022, the Guidelines to Strengthen the Governance over Ethics in Science and Technology were issued by the General Office of the Communist Party of China Central Committee and the General Office of the State Council. This document details the basic principles and requirements of Chinese science and technology ethics governance, management systems and mechanisms, and the NSTEC’s responsibilities and functions. It also states that the government should strengthen education related to science and technology ethics and related information dissemination [33]. The Guidelines mark a new stage of governance over ethics in science and technology in China.

**Figure 3:**
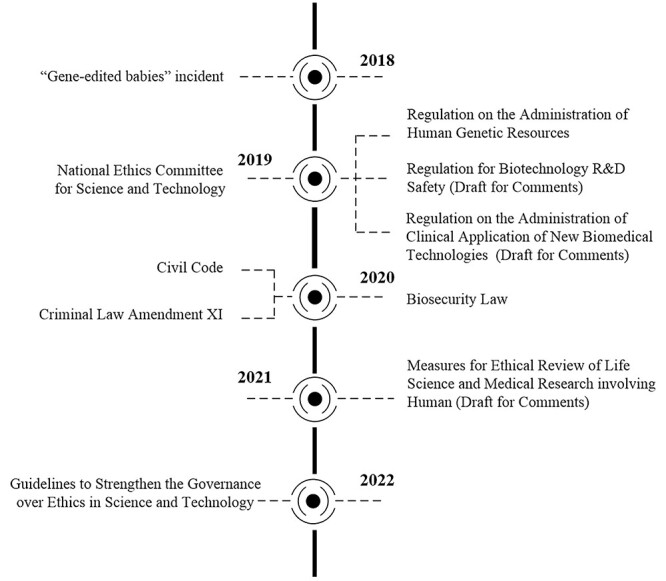
Recent developments in regulations in China.

China has gradually promoted the formulation of laws and regulations in the life science and technology field, and several laws and regulations have been drafted or revised (Figure 3). For instance, the Chinese Civil Code (CCC) was issued in May 2020. The CCC states that medical and scientific research activities concerning human genes and embryos, among others, shall be performed according to laws and administrative regulations and relevant provisions outlined by the state without endangering human health, violating moral principles, or damaging public interest [34]. According to this provision, those who engage in relevant scientific research and medical activities that contravene ethics and morality in China will be considered to have violated personal rights and can thus be subject to civil liabilities. In addition, China has promulgated the Criminal Law Amendment XI, which clearly prohibits human cloning and human germline genome editing for clinical purposes [35].

In terms of general law, the China Biosecurity Law (CBL) was promulgated in 2020. The CBL outlines the risk prevention principles of biosafety regulation [36] and the biosafety risk prevention and control system [37]. The CBL requires that research, development, and application of biotechnologies conform to ethical principles. It also emphasizes that scientific research institutes, medical institutes, and other enterprise and business units should strengthen biosecurity awareness and ethical awareness among students and practitioners.

The NHC also issued the Regulation on the Administration of Clinical Application of New Biomedical Technologies (Draft for Comments) and solicited opinions from the public in 2019 [38]. In accordance with this proposed regulation, before any clinical research and application of a new biomedical technology, such as gene editing, commences, approval should be obtained from the government. This proposed regulation divides new biomedical technologies into three categories with low, medium, and high risks; gene editing involving the alteration or regulation of genetic material expression is categorized as new biomedical technologies with high risk [39]. Moreover, clinical research on new high-risk biomedical technologies is regulated directly by the NHC. This regulation also explicitly stipulates that clinical research on new biomedical technologies should pass both academic and ethical review, and the clinical application of technologies should pass technical assessment and ethics review [40]. In case of major ethical concerns regarding new biomedical technologies, clinical research should be prohibited [41].

In 2019, the MOST issued the Regulation for Biotechnology R&D Safety (Draft for Comments) [42]. This regulation aims to regulate scientific research activities, prevent some organizations and individuals from committing serious violations of social ethics or bioterrorism in biotechnology research and development activities, and avoid direct or indirect biosafety hazards. Moreover, the Measures for Ethical Review of Life Science and Medical Research involving Human (Draft for Comments) has been revised and was released in 2021 [43]. This legislation is no longer confined to the field of biomedicine and has a broader scope extending to life science and medicine. The latest developments in life science and medicine indicate that the basic principles of ethical review, informed consent, ethical review system and mechanism have been adjusted or amended in the aforementioned measures.

Given the “gene-edited babies” incident, the proactionary principle is inappropriate for China, especially given the rapid development of emerging technologies. In this regard, China’s governance of life science compensates for the weaknesses in the regulation of human germline genome editing and the regulation of relevant applications as well as for ethical oversights in other life science research fields. Importantly, the philosophy and approach of the governance of science and technology in China, especially with regard to biotechnology research and applications, has changed gradually from proactionary to precautionary.

## Implications and recommendations

The matter of regulatory systems for human germline genome editing is not specific to a jurisdiction but is applicable globally [44]. Considering the scientific risks and unresolved ethical or social concerns associated with human genome editing, a balance between the benefits of gene editing technology and possible risks should be established in jurisdictions, including China. Currently, the clinical application of human germline genome editing is prohibited worldwide, but basic research is allowed under rigorous regulation. This is also being practiced in China, where recent changes in the regulations for biotechnology and its applications have compensated for substantial regulatory legislative deficiencies. However, further improvements in China’s current regulatory system remain necessary. These improvements must consider both the regulatory system and a comparative perspective.

## Coordination of legislation and agencies

After the “gene-edited babies” controversy, China has rapidly established a legal and regulatory system for the life science and technology field; this system comprises basic and general laws, administrative regulations, department rules, and regulatory documents. However, China still does not have a unified specific law or regulation governing human germline genome editing. Provisions concerning governance are distributed across various laws, regulations, department rules, and regulatory documents. As a result, the plethora of complicated legal documents may be difficult to comprehend by researchers, the public, and even legal professionals, which reduces the effectiveness of the regulatory system to some extent. By contrast, in many technologically developed jurisdictions, human germline genome editing is regulated by a unified law, such as the United Kingdom’s Human Fertilization and Embryology Act [45], Germany’s Embryo Protection Act [46], Canada’s Assisted Human Reproduction Act [47], and Australia’s Prohibition of Human Cloning for Reproduction Act [48].

Although a comprehensive regulatory system in the field of life science and technology has been initially established, relevant provisions in the Civil Code, Criminal Law Amendment XI, Biosecurity Law, and other laws that have been newly promulgated, and specific rules are still lacking. Moreover, the original department rules and regulatory documents overlap and lack convergence because of multiple political challenges. Although the NSTEC has been established, China’s current regulatory regime involves numerous national agencies with unclear authority and responsibilities; thus, which authority is responsible for regulating and managing human germline genome editing remains unclear. To solve the aforementioned problems, a specific law or regulation for governing activities in this field and for clarifying the regulatory scheme for biotechnologies, including CRISPR technology, involving human embryos or gametes should be developed in China. The functional boundaries between regulatory authorities should be clear in the relevant laws and regulations, and effective legal supervision should also be also ensured.

## Ethics review system

The ethics review system established in China is somewhat problematic. The system is based on three levels: the national level, provincial level, and local research or medical institution level. However, the supervision and management of ethics review work is not centralized to a single administrative department (e.g. the NHC) nor is it assigned to several ethics review committees at a high level; instead, the ethics review is delegated to an ethics review committee at the institutional level. In other words, researchers in China aiming to conduct research or clinical trials of gene editing technologies only require approval from the local institutional ethics review board. In this regard, if the research has major ethical issues, such as genetic editing of embryos for reproductive purposes, local ethics committees may not be able to address these challenges; this may lead to a situation in which major ethical and social issues have not been resolved or incidents at the institutional level may arise, but the research has already been initiated or has even been completed. Expecting institutional or local ethics review committees to address major ethical issues is unreasonable. Moreover, research involving human germline genome editing may be conducted covertly, and considerable information asymmetry may exist between the researchers and regulators. The researchers may bypass the ethical review to conduct such activities. In this case, regulatory authorities are unable to appropriately supervise these studies, and they do not become aware of the major ethical issues in a timely manner.

Although almost every aforementioned department rule and regulatory document in China requires the establishment of ethics committees, these organizations do not have any authority, granted by laws, to review and approve research. Even if relevant guidelines, such as the Measures for Ethical Review, indicate that the ethics review is mandatory, and without the approval of the ethics committee, research or clinical trials cannot be conducted, the corresponding punishment may be insufficient. Thus, the ethical review requirement exists in name only. By comparison, conducting a similar study or trial in the United States usually requires reviews by the National Institutes of Health’s Recombinant DNA Advisory Committee, the Food and Drug Administration, and the local institution [49]. In the United Kingdom, a central authority at the national level, the Human Fertilization and Embryology Authority (HFEA) [50], oversees such research and clinical trials. The decentralization of the ethical review in China may result in a lack of uniform standards across committees within the country, and local ethics committees cannot address major ethical issues.

The establishment and operation of the NSTEC may help reduce the fragmentation of regulations across departments and may address the lack of uniform standards across the country’s committees. However, the NSTEC does not review specific research projects; it is responsible for guiding, coordinating, and promoting the construction of an ethics governance system for national science and technology [33]. In addition, the Guidelines to Strengthen the Governance over Ethics in Science and Technology explicitly propose to “explore the establishment of professional science and technology ethics review centers, regional science and technology ethics review institutions,” and “gradually establish a mutual recognition mechanism for the results of science and technology ethics review” [33]. Under the leadership of the NSTEC, the ethics review standards employed across China might be gradually unified, ethics rules in the biotechnology field might be developed, and the power and responsibility of ethics review institutions might be clarified in the near future.

## Public engagement and education

As mentioned earlier in the text, the Chinese public is extremely concerned about the notion of editing the genes of babies, with high social involvement. However, although these discussions have a lot to do with the occurrence of the “gene-edited babies” incident, on the whole, the Chinese public’s enthusiasm for science and technology ethics governance is not high, and participation is low [51, 52]. In fact, public engagement, including public debate, plays a vital role in the governance of science and technology ethics. Public engagement is conducive to the communication of pluralistic governance subjects, the coexistence of pluralistic ethical views and the clarification of ethical issues, fair and open governance decisions and the acceptability of governance decisions [52]. In the United Kingdom, public engagement, discussion, and pertinent influence on legislative policies regarding the “14-day rule” for human embryo research and mitochondrial replacement technology are high [53, 54]. Against this backdrop, the channels of public engagement in China may be broadened by establishing specific channels for public participation, such as public opinion survey, public consultation, or public representatives’ attendance at relevant ethics governance seminars during the formulation of laws and policies concerning science and technology.

In China, public awareness and education regarding science and technology ethics are particularly important. The recently released Guidelines to Strengthen the Governance over Ethics in Science and Technology require the promotion of ethics education in science and technology, the institutionalization of ethical training programs and the popularization of ethical codes, as well as responsibility of news media [33]. Moreover, scientific research in the field of life science and technology should be open and transparent. However, these provisions remain relatively abstract; thus, publicity and education efforts are still urgently required.

## Conclusions

With advances in genome editing technology, this technology is being increasingly applied to research involving humans. However, because human germline genome editing has potential effects on later generations, it entails ethical, legal, and social considerations beyond those of somatic genome editing. After the “gene-edited babies” incident, ethical issues in the research and application of life sciences and ethical governance have gained prominence in China. The ethical governance in this field in China has shifted toward the precautionary approach. Moreover, the legislative work in the field has been implemented at a more rapid pace, and the ethics review and supervision have been strengthened. However, these efforts are gradual and will not be completed overnight. Despite the recent changes, we believe that the regulatory system for germline genome editing requires further improvement in three aspects: coordination of legislation and agencies, establishment of an ethics review system at high levels, and public participation and education.

## Author contributions

Yaojin Peng and Jianwei Lv should be considered joint first author.

## Conflict of interest

The authors have declared that no conflict of interest exists.

## Footnotes

1 There are two reasons for choosing this period to conduct the data analysis. Firstly, the big data analysis platform that we used generally selects data within one year for analysis, since the acquisition of big data during this period is more comprehensive and accurate. Secondly, the public opinion analysis is to obtain the public’s recent attitudes and opinions on human germline genome editing, rather than that of the “gene-edited babies” incident. Indeed, the influence between each issue we cannot deny and should be taken into consideration.
